# The Relationship between the Concentration of Salivary Tyrosine and Antioxidants in Patients with Oral Lichen Planus

**DOI:** 10.1155/2019/5801570

**Published:** 2019-11-29

**Authors:** Dagmara Darczuk, Wirginia Krzyściak, Beata Bystrowska, Barbara Kęsek, Dorota Kościelniak, Maria Chomyszyn-Gajewska, Tomasz Kaczmarzyk

**Affiliations:** ^1^Chair of Periodontology and Clinical Oral Pathology, Jagiellonian University Medical College, Montelupich 4, 31–155 Krakow, Poland; ^2^Department of Medical Diagnostics, Jagiellonian University Medical College, Medyczna 9, 30–688 Krakow, Poland; ^3^Department of Toxicology, Chair of Toxicology, Jagiellonian University Medical College, Medyczna 9, 30–688 Krakow, Poland; ^4^Department of Periodontology and Oral Medicine, Jagiellonian University Medical College, Montelupich 4 31–155 Krakow, Poland; ^5^Department of Pediatric Dentistry, Institute of Dentistry, Jagiellonian University Medical College, Montelupich 4, 31–155 Krakow, Poland; ^6^Department of Oral Surgery, Jagiellonian University Medical College, Montelupich 4, 31–155 Krakow, Poland

## Abstract

The diagnosis of oral lichen planus (OLP) is based on clinical examination and histopathological criteria. Noninvasive diagnostics of saliva may be considered as a confirmation of OLP diagnosis and a potential alternative to an invasive method. The objective of the present study was to evaluate the relationship between the level of tyrosine (Tyr) as well as antioxidants like uric acid (UA) and glutathione peroxidase (GPx) activity in the saliva of patients with OLP in comparison with the control group (healthy subjects without any oral changes). A total of 40 patients with OLP and 40 healthy volunteers were selected for the study based on the modified WHO diagnostic (clinical and histopathological) criteria. High-performance liquid chromatography (HPLC) was performed for Tyr concentration, while GPx activity and uric acid levels were determined by a colorimetric method. The concentrations of Tyr, UA, and GPx activity were statistically lowered in OLP patients compared to the control group. All examined parameters correlated strongly and positively with each other. Mean values of salivary UA concentrations differed between the groups of OLP patients (reticular and erosive forms) and controls (206.66 vs. 196.54 vs. 218.49 *μ*mol/L, respectively, *p* = 0.001). A similar trend was demonstrated in salivary Tyr concentration which differed statistically between the study and control groups (0.08 vs. 0.07 vs. 0.13 *μ*mol/L, respectively, *p* = 0.001). Determining of a relationship between the concentrations of Tyr, UA, and GPx activity may be useful in the prognosis of OLP. The HPLC method may be employed, as an additional noninvasive diagnostic procedure to screen OLP patients, during the routine diagnostics of salivary biochemical parameters such as aromatic amino acids.

## 1. Introduction

One of the most commonly encountered lesions of the oral mucosa is oral lichen planus (OLP) which affects 0.1–4% of the adult population. It is a chronic inflammatory disease of the oral mucosa of unknown etiology. OLP is considered a potentially malignant disorder with an approximately 1% risk of developing oral squamous carcinoma [1]. Several clinical presentations of OLP are recognized, with the most common being reticular and erosive types. The reticular form presents as fine white lines (known as Wickham's striae) with an erythematous border. The striae are usually asymptomatic and located symmetrically on the buccal mucosa, on the tongue and gums. In contrast, erosive OLP is predominantly associated with pain and oral discomfort and presents as a mixture of ulcerated and erythematous areas, surrounded by striae. Typically, one patient may have a different presentation of OLP transforming from one pattern to another over time with unknown reason [2]. Interestingly, there is a significantly higher risk of malignant transformation in the course of erosive OLP in comparison with its reticular form (33% vs. 13%, respectively), and hitherto, an invasive biopsy remains the gold standard for diagnosis as well as monitoring of OLP and other potentially malignant disorders [3]. Hence, researchers have tried to investigate alternative, noninvasive methods of monitoring potentially malignant oral disorders.

It has been suggested that in OLP, oxidative stress can initiate local tissue damage [4] and modify the clinical form of the disease. This stems from the fact that many transcription factors are related to the induction of oxidative stress in OLP, including Nrf2, NF-*κ*B, Notch, p-53, and AP-1. Their contribution translates into the regulation of the expression of genes encoding compounds responsible for detoxification of free radicals (enzymatic and nonenzymatic antioxidants), restoration of oxidative-antioxidative balance by these compounds, and control of further cell functions, such as proliferation or apoptosis [5]. The extent to which these changes will occur and their further impact on clinical manifestations of OLP (reticular or erosive type) depends on the local level of salivary antioxidants (antioxidants that can defend against oxidative stress) [6]. Antioxidative enzymes present in mammalian cells, such as superoxide dismutase (SOD), catalase (CAT), or glutathione peroxidase (GPx), as well as nonenzymatic antioxidative systems which include, among others, urinary acid (UA) or vitamins A and E [7] may significantly affect these processes. The results of many studies indicate that UA plays an important role in the removal of free oxygen radicals as the dominant nonenzymatic antioxidant in the body [8]. Its salivary level has been proven to be closely related to blood content [9].

A number of studies have appraised the importance of monitoring oxidative stress parameters in the saliva of OLP patients [10–13]. Their results indicating a decline in the elements of antioxidative defense (decrease in levels of GSH, FRAP, CAT, and SOD) and intensification of oxidative stress (increase in levels of malonyldialdehyde or 8-hydroxy-2′-deoxyguanosine) compared to the healthy individuals confirm escalation of oxidative/nitrosative stress in patients with OLP [11, 14–16]. In our previous report, we demonstrated that the values of antioxidant potential and reduced glutathione are approximately 21% lower in the reticular form of OLP and by approximately 40% in its erosive type as compared to controls [11]. Likewise, we found TBARS determinations to be significantly different between both forms of OLP [11]. However, no further data are available to explain the observed changes with respect to the clinical OLP phenotype.

**Figure 1:**
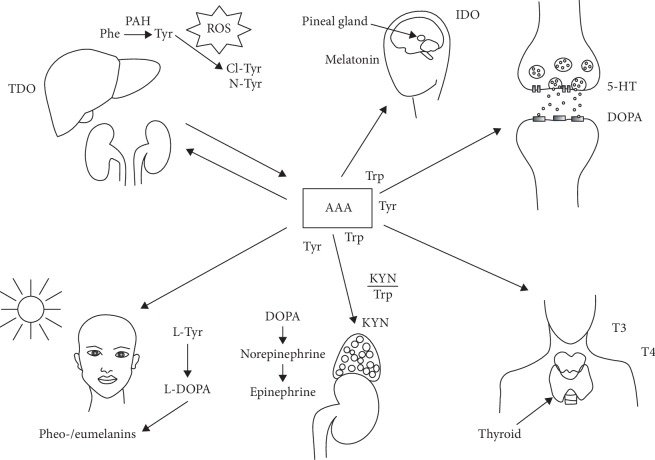
The role of aromatic amino acids (AAA) in the organism and induction of tyrosine derivatives (3,4-dihydroxyphenylalanine (DOPA) and 2,5-dityrosine) by reactive oxygen species. Phenylalanine (Phe) is converted to tyrosine (Tyr) in the liver and kidneys with the participation of phenylalanine hydroxylase (PAH). Tyr is used for the synthesis of catecholamines (dopamine, epinephrine, and norepinephrine) in the adrenal medulla and for the synthesis of pheo- and eumelanins in skin melanocytes, the first stage of which is the formation of L-DOPA. An elevated L-DOPA/L-Tyr ratio occurs in patients with malignant melanoma and local lymph node metastases. Tyr is also the starting compound for the synthesis of thyroid hormones—thyroxine (T4) and tri-iodothyronine (T3). In addition, Tyr derivatives such as 3-Cl-D-Tyr or 3-N-D-Tyr may be formed under oxidative, nitrifying, and chlorinating stress conditions. Tyr catalyzes the reaction that converts Trp to kynurenine (KYN). IDO (indoleamine-2,3-dioxygenase) and TDO (tryptophan-2,3-dioxygenase) are induction enzymes that are activated under stressful situations, among others under the proinflammatory cytokines.

It has been proposed that the level of salivary tyrosine, classified in a group of aromatic amino acids (AAA), may be used to discriminate clinical manifestations of OLP. *In vivo*, tyrosine (Tyr), and more specifically its derivative, e.g., 3-nitrotyrosine (3-NT), undergoes free radical modifications, making it a promising biomarker for the intensification of oxidative/nitrosative stress (Figure 1) [17–19]. With regard to ongoing free radical stress observed through apparent decrease in the key antioxidant enzymes (GPx,SOD, and CAT), Tyr nitration can block the mechanism associated with their recompensation (restoring their original antioxidant functions). Hence, some antioxidant enzymes (including manganese superoxide dismutase, MnSOD) under conditions of increased oxidative/nitrosative stress temporarily lose their original functions (associated with defense against free radicals) and, for the time being, become inactive forms of enzymes. This contributes to the generation or exacerbation of already existing oxidative stress [20]. The molecular mechanism of the observed changes relates to changes in the activity of intracellular signaling pathways mediated by, among others, tyrosine kinases, i.e., MAPK and PI3K or transcription factors (i.e., Nrf2, AP-1, NF-*κ*B, and p53), maintaining normal functions of proteins involved in the preservation redox balance in cells, i.e., enzymes producing reactive oxygen and nitrogen species (RONS) and system component antioxidants [21]. These disturbances may cause further pathological changes, resulting in apoptosis of keratinocytes or their degeneration, which is more frequently observed in the erosive form of OLP (more prone to malignancy) than in its reticular counterpart. Interestingly, abnormalities in Tyr levels may change the clinical phenotype of the disease as a result of damage to the extracellular matrix in OLP [22], which would explain the somewhat subjective changes in clinical signs and symptoms of OLP.

Monitoring of salivary levels of antioxidant system parameters is a noninvasive alternative to surgical intervention, i.e., taking a biopsy for histopathological examination. Previously published studies have focused on the determination of oxidative stress in the general group of patients with OLP, regardless of the clinical phenotype of the disease. In the current study, we attempted to determine potential markers which enable assessing the level of salivary antioxidative defense and verifying any oxidative-antioxidant imbalance associated with the clinical phenotype of OLP.

Hence, the aim of the current study is to assess the usefulness of salivary amino acid determination in discrimination of Tyr levels between patients with OLP and healthy individuals and to determine its relationship with salivary antioxidative defense indicators represented by GPx and UA, with the intention of establishing a distinction between reticular and erosive OLP forms.

## 2. Materials and Methods

### 2.1. Patient Recruitment

The study was performed in the Department of Periodontology and Oral Medicine, Institute of Dentistry, Jagiellonian University Medical College, Krakow, Poland. The recruitment of patients was carried out between May 2016 and December 2018. The number of study participants totaled 40. All participants gave written, informed consent. The study protocol was approved by the Bioethics Committee of the Jagiellonian University in Krakow, Poland (No. KBET/112/b/2014). The study was performed in accordance with the Helsinki Declaration of 2008.

The criterion for inclusion was the clinical diagnosis of reticular or erosive form of OLP. Pregnant women, patients treated for systemic or local diseases, patients supplemented with antioxidants (vitamins, preparations containing antioxidants, i.e., ointments and extracts) within the last three weeks, patients with any inflammatory lesions of the oral mucosa, patients with history of oral malignancy, patients chewing gum, and those treated for OLP in the last 3 months were excluded from the study.

Clinical diagnosis of reticular OLP was made in the cases of asymptomatic oral lesions presenting with white lacy lines surrounded by an erythematous border. Twenty-six patients with reticular OLP were recruited to the study group. The lesions were located mainly on the buccal mucosa and less frequently on the tongue or the gums.

In turn, clinical diagnosis of erosive OLP was made in the cases of symptomatic oral lesions presenting with erosions or ulcerations within erythematous changes surrounded by white lacy lines. Fourteen patients with erosive OLP were recruited to the study group. Erosive OLP lesions were mainly located on the buccal mucosa, edges of the tongue, or simultaneously in both sites.

The tentative clinical diagnosis of reticular or erosive OLP was made by a dentist with experience in oral medicine. The clinical diagnosis was subsequently confirmed by a histopathological examination as per the modified WHO diagnostic criteria [23].

Forty healthy individuals without any lesions of the oral mucosa were recruited to the control group.

### 2.2. Collection and Preparation of Saliva

Patients were asked to avoid eating meals and consuming any liquids 2 hours before the examination. Before the saliva collection, patients rinsed their mouth thoroughly with a minimum of 10 mL of deionized water. They sat comfortably with their eyes open and their head slightly tilted forward to facilitate the collection of saliva from the floor of the mouth. Saliva samples were collected between 9 and 11 AM with the subjects chewing a cotton swab devoid of any saliva-stimulating agent (to avoid interference) for 5 min. The swab was then placed in a sterile plastic centrifuge tube provided by the manufacturer (Salivette Sarstedt Germany System). The samples were stored at 4°C and transported on ice. Material preparation for the examination started no later than 2 hours after sampling. The saliva samples were centrifuged for 10 min at 900 rpm at 4°C, and the resulting filtrate was transferred to sterile 1.5 mL microtubes, which were then centrifuged at 10,000 rpm at 4°C for 10 minutes to remove debris (insoluble components, cells, and impurities). A part of the saliva was transferred to 1.5 mL microtubes with the addition of 15 *μ*L of 0.15 M solution of butylated hydroxytoluene in ethanol per 1 mL of saliva, in order to prevent lipid peroxidation during storage of the samples. A part of the saliva was deproteinized using trichloroacetic acid, 30 *μ*L of which was added to 90 *μ*L of the material, vortexed, and centrifuged for 5 min at 12,000 rpm. The supernatant was transferred to a separate tube placed on ice. The acidic clear solution was stored at -80°C until HPLC analysis (no more than 2 months).

### 2.3. Determination of Tyrosine

All chemical solvents and standards were of analytical grade. Acetonitrile was delivered from Merck (Darmstadt, Germany) and formic acid from POCh (Katowice, Poland). HPLC ELITE LaChrom VWR (Merck) instrumentation consisted of the following components: a pump series L-2130 in gradient mode, an autosampler series L-2200 with a 100 *μ*L injection loop, Column Oven series L-2350, and the diode array detector (DAD) series L-2455. The chromatographic separation was performed in the gradient mode with a Discovery RP 18 column (250 × 4.6 mm I.D., 5 *μ*m particle size) from Sigma-Aldrich (Supelco).

The mobile stage consisted of the phase A which was 0.1% *v*/*v* formic acid (HCOOH) in water and the phase B which was acetonitrile (0.050% TFA in acetonitrile/water 80 : 20 *v*/*v*). The flow rate increased from 0.6 mL/min to 1.2 mL/min for 11minutes and then decreased to 0.6 mL/min. The gradient was programmed as follows: 6% B for 11 min, then increased to 14% B for 3 min, and then decreased to 6% B. Temperature of the column was equal to 15°C. The applied gradient was linear from 0 to 55% in 11 min, at a flow rate of 1.2 mL/min.

The DAD was set at a wavelength of 274 nm. Mass spectra were collected every 3 ms in the positive ion mode. MS spray voltage was 4.50 kV and the capillary temperature was 250°C.

### 2.4. Determination of GPx

Determination of GPx in saliva was performed using the method proposed by Paglia and Valentine [24] with the modification of Lawrence and Burk [25] using t-butylhydroperoxide as a substrate. GPx is a catalyst for GSH oxidation. In the presence of glutathione reductase (GR) and NADPH, the oxidized GSH (GSSG) is converted to the reduced form (GSH) with concurrent NADPH oxidation to NADP^+^.

(1) $$\begin{array}{c} {\text{H}}^{+}+\text{GSSG}+\text{NADPH}\to 2\text{GSH}+{\text{NADP}}^{+} \end{array}$$

The GPx enzyme catalyzes the oxidation of GSH to a form that reacts with NADPH and produces the oxidized form of NADP and two reduced GSH molecules.

As much as 50 *μ*L of saliva was mixed with 1.5 mL of a reaction mixture containing 2 mM *β*-NADPH, 1 M sodium azide, 100 mM reduced GSH, and 100 U/mL GR (the GPx enzyme catalyzes the oxidation of GSH to a form which reacts with NADPH and produces the oxidized form of NADP and two GSH molecules). The reagents were mixed by inversion, and 50 *μ*L of 5 mM H_2_O_2_ was added as an initiator of the reaction, while a decrease in absorbance at *λ*_max_ = 340 nm was measured. All measurements were performed using the POLARstar plate reader from BMG Labtech.

### 2.5. Determination of UA

The level of UA in saliva was measured colorimetrically using a commercial enzyme kit (cat. no. 1–3802, Salimetrics, CA, USA) according to the manufacturer's instructions. UA is converted by uricase into allantoin and hydrogen peroxide which oxidizes 4-aminophenazone to sodium N-ethylmethaniline propanesulfonate under the influence of peroxidase, producing a red chromogen whose color intensity is proportional to the amount of UA present in the saliva sample. The working reagent (1 mL) and saliva sample or blank standard (0.020 mL) were mixed and incubated for 5 min at 37°C. The absorbance values of the standard, test, and blank samples were read at a wavelength of *λ*_max_ = 546 nm.

### 2.6. Statistical Evaluation

The calculation of the results was based on the regression equation for the 10 series of standard curves in *μ*mol/mL. Thus, the obtained results were compared with the results obtained using an external standard (L-Trp and D-Tyr). Comparisons of quantitative variables in two groups were done with Student's *t*-test (in case of normal distribution in both groups) or with the Mann-Whitney test (otherwise). Comparisons of quantitative variables in more than two groups were performed using ANOVA (in case of normal distribution in each group) or with Kruskal-Wallis test (otherwise). Fisher's LSD test (in case of normal distribution in each group) or Dunn's test (otherwise) was used as *post hoc* procedure. Correlations between quantitative variables were assessed with Pearson's test (in case of normal distribution of both variables) or Spearman's test (otherwise) correlation coefficient. The strength of association was judged with Hinkle's scheme: ∣*r*∣ ≥ 0.9—very strong, 0.7 ≤ ∣*r*∣ < 0.9—strong, 0.5 ≤ ∣*r*∣ < 0.7—moderate, 0.3 ≤ ∣*r*∣ < 0.5—weak, and ∣*r*∣ < 0.3—very weak [26]. Normality was assessed with the Shapiro-Wilk test. Analyses were conducted at 0.05 level of significance. For all statistical analyses, the R software, version 3.6.1, was used.

## 3. Results

Basic descriptive statistics for Tyr concentration (*μ*mol/mL), GPx activity (mU/mL), and UA concentration (*μ*mol/L) in saliva of the study participants are presented in Table 1.

Due to the fact that there were no statistically significant differences in terms of gender for the studied parameters (*p* > 0.05; Table 1), subsequent analyses were performed without division into groups of females and males.

**Figure 2:**
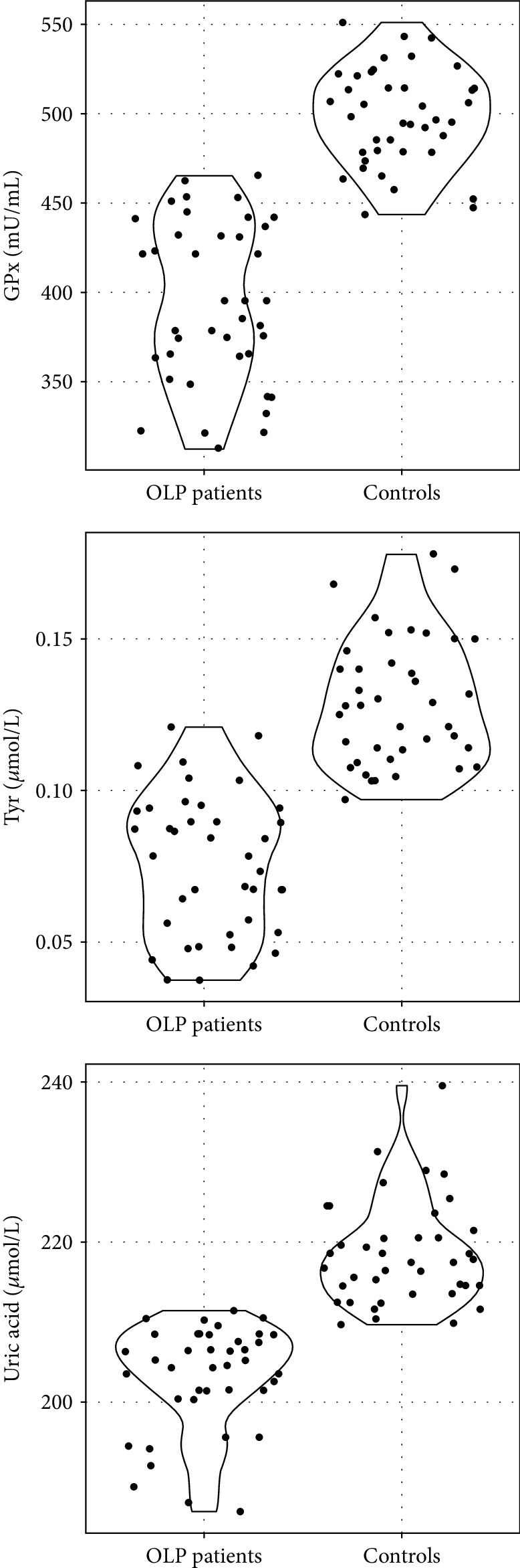
Violin plot representing tyrosine (Tyr), uric acid (UA), and glutathione peroxidase (GPx) activity in the group of OLP patients and controls. The dots on the graph represent individual results in each group.

For each of the tested parameters, i.e., Tyr, GPx, and UA, the differences between the group of patients with OLP and the control group were statistically significant, as presented in Table 2. All tested parameters were higher in the control group than in patients with OLP. These differences are illustrated in Figure 2.

**Figure 3:**
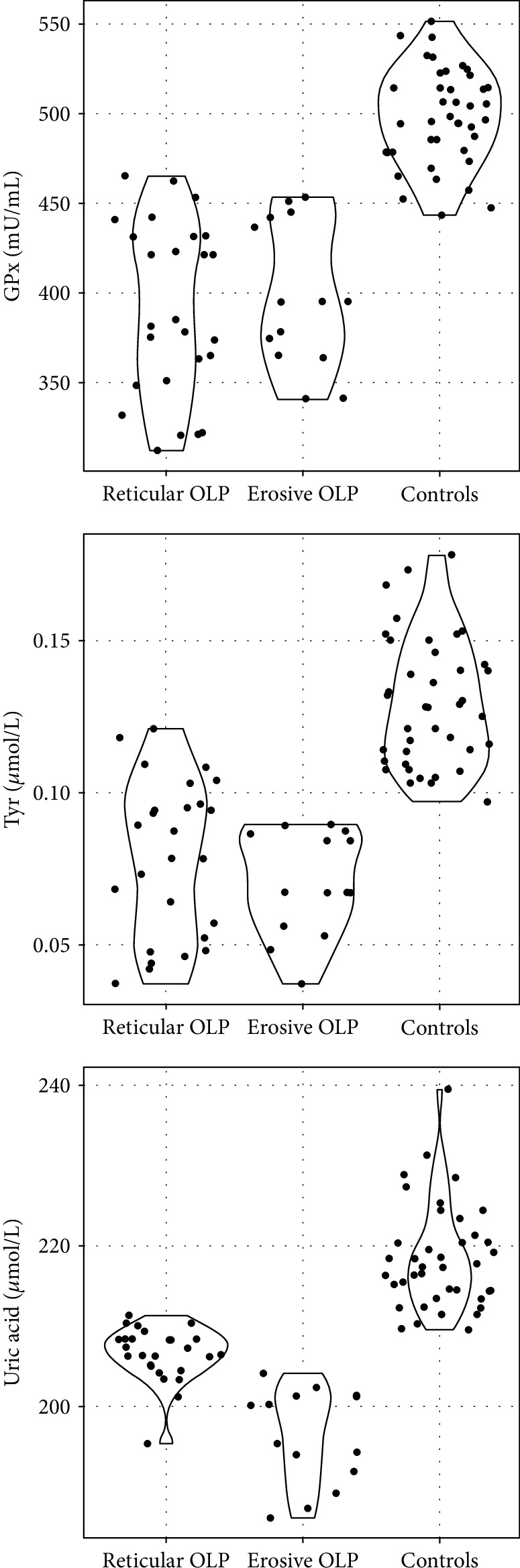
Violin plot representing tyrosine (Tyr), uric acid (UA), and glutathione peroxidase (GPx) activity in the subgroups of patients (erosive and reticular OLP) and controls. The dots on the graph represent individual results in each group.

For each of the tested parameters, the differences between the groups were significant (Table 2, Figure 2). A *post hoc* analysis was performed to assess the nature of dependencies. Levels of GPx and Tyr were significantly higher in the control group than in both groups of patients with OLP which did not differ from each other. Concentration of UA, however, was significantly higher in the control group than in the reticular group, which, in turn, was significantly higher than in the erosive group as presented in Table 3 and Figure 3.

**Figure 4:**
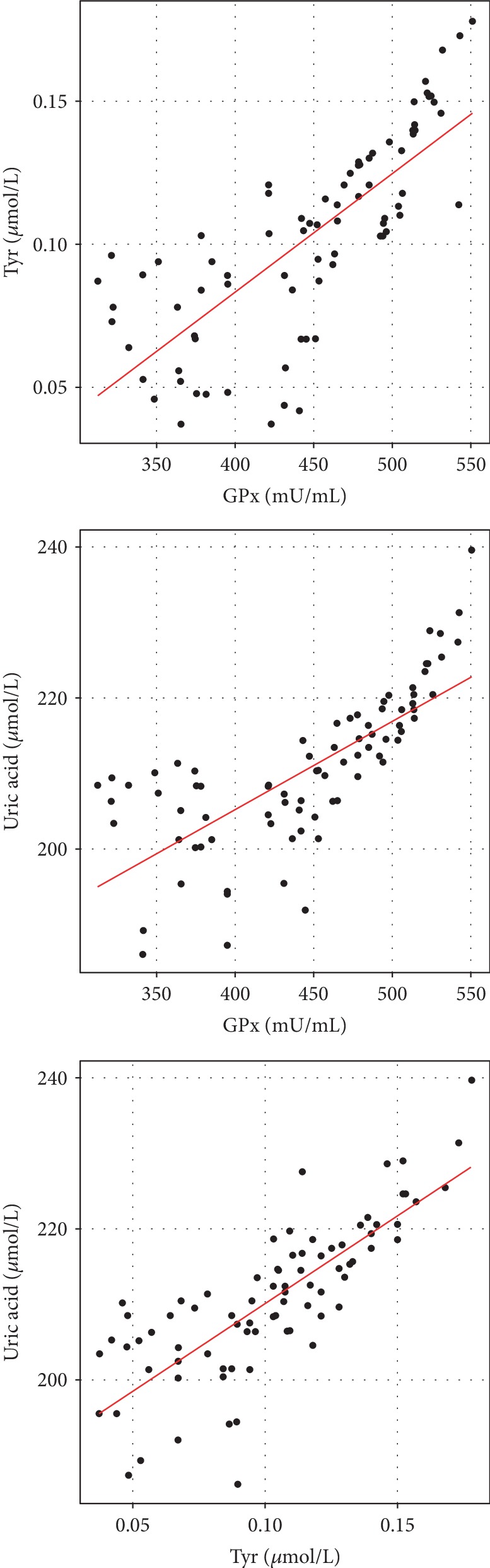
Scatter (correlation) charts depicting the relationships between the studied parameters, i.e., tyrosine, glutathione peroxidase, and uric acid. Individual observations are shown in groups.

All three parameters correlate significantly and positively (*p* < 0.05); the higher the value of one of them, the higher the values of the remaining ones as illustrated in Table 4 and Figure 4.

**Figure 5:**
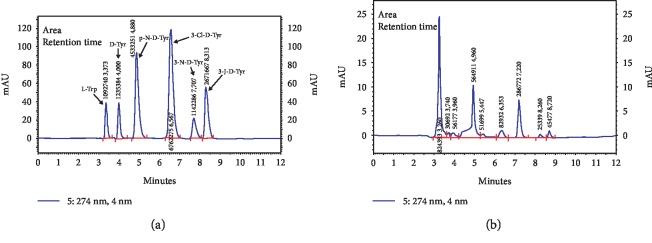
Sample chromatogram (HPLC) for tyrosine and its derivatives: (a) calibration curve and (b) examined sample.

The results of Tyr concentration obtained using the HPLC method correlated significantly (strong positive correlation) with the results of GPx activity and UA concentrations in saliva (Figure 5).

## 4. Discussion

### 4.1. Glutathione Peroxidase (GPx)

It is increasingly common practice that the research into new biomarkers of oral diseases, including OLP, is focused on the determination of disease predictors in a noninvasively derived material, such as saliva, due to the ease of collection and the fact that it represents a good alternative to the invasive method, i.e., tissue biopsy [27]. Collection of saliva is a simple procedure which can be carried out as often as necessary, without any complications, such as pain sensations, that may follow invasive testing such as blood or tissue collection. The documented correlation between the aforementioned parameters in the saliva, blood, or tissues determines the use of saliva as a biological fluid reflecting the intensification and rate of pathological changes with local nature (notably in the case of oral cavity diseases) [14, 28]. Thus, this study focused on the determination of the levels of selected antioxidants (the enzymatic antioxidant glutathione peroxidase (GPx) and the nonenzymatic antioxidant with its major representative uric acid (UA)) in the saliva of patients with OLP.

The present study also investigated the levels of tyrosine, one of the most important AAA in the human body regulating the cellular metabolism necessary for the synthesis of endogenous proteins. Specifically, we verified the hypothesis that alterations in concentrations of the above parameters in saliva may reflect the capacity of the human body to prevent the effects caused by reactive oxygen and nitrogen species (ROS/RNS) generating oxidative/nitrosative stress associated with the pathogenesis of OLP [29].

In the study by Rekha et al. [30], the activity of GPx determined for 40 patients with OLP, the levels of salivary GPx (0.06 ± 0.03) in patients with OLP were significantly lower than in controls, which is in line with our results. Likewise, in the study by Totan et al. [14], GPx activity was similar to that in our study. The mean GPx value in the saliva of patients was significantly lower than that of controls (28.16 ± 11.95 U/mg albumins vs. 29.97 ± 17.78 U/mg albumins, respectively). In the current study, a similar decrease in GPx activity was observed in OLP as compared with controls, although a comparison of absolute GPx activity values was impossible due to the material used for testing (higher values in the serum than in plasma) and the different method and period of material storage. In none of the cited studies was there an addition of butylated hydroxytoluene (BHT) to limit *in vitro* lipid or protein peroxidation. Furthermore, Rekha et al. [30] provided neither the units in which the results were calculated nor the literature references for the measurement method, despite the fact that the authors used the spectrophotometric method and sample to reagent ratios similar to ours [30].

The statistical analyses performed in this study further demonstrated an upward trend in mean GPx activities in patients with the reticular form in comparison to the erosive oral lichen planus form in relation to the control group. The GPx values were lower in those patients with the reticular as opposed to the erosive form and lower in OLP than in controls without significant differences within sexes.

The observed changes can be explained by the higher compensation of GPx in patients with the erosive form as compared with the reticular form, following the chemopreventive action of the enzyme in the course of more intense oxidative changes. This can also be explained by the fact that the higher activity of GPx in its erosive form may suggest a reduced likelihood for the attack of oxygen free radicals in the site of enzyme action. The obtained results are in accordance with those of other studies in terms of the drop in GPx activity in OLP (without the clinical differential of the disease phenotype). Considering the fact that no published results from studies exist, which would differentiate between GPx activity based on the clinical form of OLP, a more detailed comparison of the obtained results (particularly of the absolute values of GPx) is not possible.

### 4.2. Uric Acid (UA)

Statistically significant differences between patients with OLP and the control group were observed in terms of the level of the salivary nonenzymatic antioxidant, that is, uric acid (UA). Uric acid being the most abundant antioxidative compound in saliva is one of the most sensitive biomarkers used to detect oxidative-antioxidative changes in the course of various diseases. Its role consists of the efficient removal of free oxygen radicals and inhibition of lipid peroxidation [31]. The present study considered only the salivary level of UA due to the linear relationship between the concentrations of UA in saliva and serum as previously reported [32]. In our study, UA was lower in the group of OLP patients than in the controls. A similar decreasing trend was observed by Totan et al. who found that the salivary level of UA in OLP patients was significantly lower than that in controls [14]. Results similar to ours were demonstrated by Battino et al. [33] and Miricescu et al. who noted a significant decrease in the salivary concentration of UA in comparison with healthy individuals (1.5–2 mg/dL in OLP vs. 3 mg/dL in controls) [34]. On the other hand, Gupta et al. found a marked reduction of UA levels in the serum of OLP patients in comparison with controls (4.058 ± 1.31 vs. 5.28 ± 0.961 mg/dL) [35]. By contrast, an increased level of salivary UA has been observed in the majority of generalized pathologic conditions such as diabetes and cardiovascular disorders, which might explain the systemic oxidative stress [36–38], as opposed to changes of local nature. The increase in UA levels in these studies may constitute a compensatory antioxidative defense system of the entire human body aimed at preventing generalized oxidative stress.

Methodological differences (namely, stimulated vs. nonstimulated saliva collection method) as well as demographic differences between the examined patients hinder the adequate comparison of the obtained uric acid concentrations. Furthermore, the present study revealed significant differences in terms of the UA level between reticulate and erosive forms of OLP. However, unlike GPx, the UA concentrations were significantly lower in the erosive form of OLP in comparison with its reticulate counterpart and controls. Similar observations were made by Gupta et al. [35], who observed that the UA level correlates with an exacerbation of OLP as compared with healthy controls. Similarly, OLP patients in remission exhibited lower UA levels than individuals with an exacerbation of the disease symptoms [35]. Some studies have also indicated a lack of differences between mean UA concentrations in patients with diabetes with different blood glucose levels, which suggests the possibility of similarities between UA concentrations in patients based on their glycated hemoglobin profiles [39, 40].

### 4.3. Tyrosine (Tyr)

Among recognized parameters used for the assessment of oxidative and antioxidative status in OLP, we tested the salivary level of Tyr. It is believed to participate in the molecular mechanism (by PI3K/Akt signaling stimulation and by NF-*κ*B and p53 transcription factor activation) constituting the basis for malignant degeneration [20]. Hence, we attempted to test the hypothesis that there are differences in its level in distinct clinical phenotype of OLP. In the case of patients with erosive oral lichen planus, a lower Tyr level was observed in comparison with patients with the reticulate form but not statistically significant. We suspect that this may constitute a negative sign indicating a gradual blockage of the antioxidative defense and a poorer response of the patients to the ongoing oxidative/nitrosative stress.

Our hypothesis may be supported by Chaiyarit et al., who showed a link between one of the Tyr modifications, i.e., 3-nitrotyrosine, and immunoreactivity; this is believed to be a biochemical marker of inflammation, particularly observed in the squamous cell cancer (OSCC) which is the one possible course of the oral lichen planus [41]. Our study was the first, so comparison of these results with other studies is impossible. It has been demonstrated that the formation of Tyr modifications may disturb the structure and physiological function of numerous proteins, intensifying oxidative stress up to the loss of their function. Detection of the presence of Tyr and its modifications in the saliva may be a promising marker differentiating the clinical disease phenotype in the future.

The present study is the first to verify *in vivo* the hypothesis of the lack of redox homeostasis due to the presence of different Tyr levels, which—together with Tyr modifications—may influence cellular homeostasis and redox signaling pathways under clinical conditions in OLP patients. In addition, our further and unpublished results determined which of the formed Tyr modifications (i.e., 3-hydroxy-tyrosine, tyrosine crossbonds, 3-nitrotyrosine, or halogenated tyrosine) have an impact on cellular functions, such as changes in the extracellular matrix or apoptosis of epithelial cells, which may be the cause for progression of the formed changes, including malignant degeneration in this group of patients. In the future, this would aid in the formulation of algorithms for procedures in patients for whom untreated erosive OLP could undergo malignant transformation.

Provision of information on the precisely modified Tyr residuals and the character of these modifications could help to understand the consequences for oxidation Tyr in clinical conditions, including determination of their influence on the cellular signaling and dysfunction of cells in different clinical forms of OLP.

It should be noted that the etiology of OLP is still unknown, despite the high incidence of the disease. OSCC is often preceded by potentially malignant disorders, such as leukoplakia, oral submucosal fibrosis, or OLP (particularly its rapidly advancing erosive form). Oxidative and nitrosative stresses play a significant role in the pathogenesis of OLP, as well as in the progression of its distinct clinical forms. Therefore, noninvasive monitoring of potential biomarkers of disease and their correlation with the clinical phenotype of the disease could play a key role in the early detection of malignancy. The available literature in this field is scarce. The preliminary conclusion from our study is that tyrosine, alongside UA and GPx, can also constitute a promising biomarker of the oral lichen planus patients based on saliva tests.

It may be a promising marker for the assessment of oxidative-antioxidative balance and monitoring of the clinical status of OLP patients.

The limitation of the present study is the small sizes of the studied groups with clinical OLP subtypes and the high level of age diversity in the study groups. Furthermore, only Tyr levels were determined, without its nitrosative modifications.

## 5. Conclusions

The results of the present study suggest that, apart from the salivary antioxidants such as UA and GPx, tyrosine should also be taken into account as a useful biomarker providing information on the oxidative-antioxidative balance in OLP. In addition, the study provides evidence for the use of the above parameters in the monitoring of clinical forms of oral lichen planus, such as the reticulate and erosive forms. There is a need for further research to confirm the presented results.

## Figures and Tables

**Table 1:** Descriptive statistics for the examined group OLP patients, i.e., reticular and erosive forms, and controls dependent on gender. P: normal distribution in all measurements, repeated measurement ANOVA test, and *post hoc* analysis results (Fisher's LSD test for couples associated); NP: no normality of distribution in at least one measurement, Kruskal-Wallis test, and *post hoc* results (Dunn's test for associated couples).

	Parameter		Males (*N* = 13)	Females (*N* = 13)	^∗^ *p*
Reticular OLP	GPx (mU/mL)	Mean ± SD	379.51 ± 44.12	403.58 ± 50.7	0.209 P
	Tyr (*μ*mol/L)	Mean ± SD	0.08 ± 0.02	0.08 ± 0.03	0.476 P
	Uric acid (UA) (*μ*mol/L)	Mean ± SD	205.91 ± 4.47	207.42 ± 1.35	0.256 P

Erosive OLP	Parameter		Males (*N* = 7)	Females (*N* = 7)	^∗^ *p*
	GPx (mU/mL)	Mean ± SD	388.64 ± 42.01	408.47 ± 39.06	0.378 P
	Tyr (*μ*mol/L)	Mean ± SD	0.07 ± 0.02	0.07 ± 0.01	0.666 P
	Uric acid (*μ*mol/L)	Mean ± SD	193.37 ± 4.84	199.7 ± 5.55	0.064
		Median	194.2	201.4	NP
		Quartiles	190.75–195.05	200.35–202	

OLP patients	Parameter		Males (*N* = 20)	Females (*N* = 20)	^∗^ *p*
	GPx (mU/mL)	Mean ± SD	382.7 ± 42.51	405.3 ± 45.95	0.115 P
	Tyr (*μ*mol/L)	Mean ± SD	0.07 ± 0.02	0.08 ± 0.02	0.655 P
	UA (*μ*mol/L)	Mean ± SD	201.52 ± 7.59	204.72 ± 5.01	0.267
		Median	203.5	206.4	NP
		Quartiles	195.32–208.43	202.25–207.72	

Controls	Parameter		Males (*N* = 20)	Females (*N* = 20)	^∗^ *p*
	GPx (mU/mL)	Mean ± SD	495.19 ± 30.28	501.19 ± 24.15	0.493 P
	Tyr (*μ*mol/L)	Mean ± SD	0.13 ± 0.02	0.13 ± 0.02	0.854 P
	UA (*μ*mol/L)	Mean ± SD	218.18 ± 7.88	218.8 ± 4.83	0.323
		Median	215.95	217.95	NP
		Quartiles	212.4–220.72	215.52–221.25	

All	Parameter		Males (*N* = 40)	Females (*N* = 40)	^∗^ *p*
	GPx (mU/mL)	Mean ± SD	438.94 ± 67.61	453.24 ± 60.58	0.368
		Median	446.30	463.75	NP
		Quartiles	380.75–495.45	421.48–498.68	
	Tyr (*μ*mol/L)	Mean ± SD	0.1 ± 0.04	0.1 ± 0.03	0.893 P
	UA (*μ*mol/L)	Mean ± SD	209.85 ± 11.38	211.76 ± 8.63	0.4 P

**Table 2:** Differences in the tested parameters (GPx, Tyr, and UA) in the saliva of OLP patients and controls. P: normal distribution in all measurements, repeated measurement Student's *t*-test, and *post hoc* analysis results (Fisher's LSD test for associated couples); NP: no normality of distribution in at least one measurement, Mann-Whitney test, and *post hoc* results (Dunn's test for couples associated).

Parameter	OLP patients (*N* = 40)	Controls (*N* = 40)	^∗^ *p*
GPx (mU/mL)			
Mean ± SD	394 ± 45.16	498.19 ± 27.2	<0.001
Median	390.25	497.40	NP
Quartiles	363.93–433.25	478.5–516.2	
Tyr (*μ*mol/L)			
Mean ± SD	0.08 ± 0.02	0.13 ± 0.02	<0.001 P
UA (*μ*mol/L)			
Mean ± SD	203.12 ± 6.55	218.49 ± 6.46	<0.001
Median	204.9	217.4	NP
Quartiles	201.15–208.4	214.25–220.72	

^∗^P—Student's *t*-test (normal distribution in groups); NP—Mann-Whitney test (nonnormal distribution in at least one group).

**Table 3:** Comparison of the glutathione peroxidase (GPx) activity and tyrosine and uric acid (UA) concentrations within groups, i.e., patients with the reticular form, patients with the erosive form, and healthy individuals without symptoms of the disease: A, B, and C. Statistically significant differences between particular groups are recorded in column 4; *p* means statistical significance at <0.05; differences between particular groups: A-C were examined by Fisher's *post hoc* test when the assumptions about normality and homogeneity of variance in groups were true or Dunn's test if otherwise.

Parameter	Reticular OLP (A) (*N* = 26)	Erosive OLP (B) (*N* = 14)	Controls (C) (*N* = 40)	^∗^ *p*
GPx (mU/mL)				
Mean ± SD	391.55 ± 48.16	398.56 ± 40.31	498.19 ± 27.2	<0.001 P C > A, B
Tyr (*μ*mol/L)				
Mean ± SD	0.08 ± 0.03	0.07 ± 0.02	0.13 ± 0.02	<0.001 P C > A, B
Uric acid (*μ*mol/L)				
Mean ± SD	206.66 ± 3.33	196.54 ± 5.98	218.49 ± 6.46	<0.001
Median	206.95	197.95	217.40	NP
Quartiles	205.22–208.5	192.62–201.47	214.25–220.72	C > A > B

^∗^P—ANOVA+Fisher LSD test (normal distribution in groups); NP—Kruskal-Wallis test+Dunn test (nonnormal distribution in at least one group).

**Table 4:** Relationship between the tested antioxidant parameters, i.e., GPx and uric acid (UA), GPx and Tyr, and between UA and Tyr. Significance level ^∗^*p* < 0.001; correlation coefficient *r*; a positive correlation direction means that the increase in GPx activity is accompanied by an increase in results of Tyr or UA. The higher the GPx activity on one variable (e.g., *x* axis), the higher the UA result on the other variable (*y* axis). The strength of the relationship between the tested parameters is high.

Parameters	Correlation			
	Coefficient (*r*)	^∗^ *p*	Direction	Strength
GPx and Tyr	0.824	*p* < 0.001 NP	Positive	Strong
GPx and UA	0.826	*p* < 0.001 NP	Positive	Strong
Tyr and UA	0.792	*p* < 0.001 P	Positive	Strong

^∗^P—Pearson correlation (normal for both parameters); NP—Spearman rank correlation (nonnormal distribution of at least one parameter).

## Data Availability

The results of the analyses of patients are covered by GDPR policy. Numerical data used to support the findings of this study are available from the corresponding author upon request.
